# Phosphorylation of cardiac voltage‐gated sodium channel: Potential players with multiple dimensions

**DOI:** 10.1111/apha.13210

**Published:** 2018-12-16

**Authors:** Shahid M. Iqbal, Rosa Lemmens‐Gruber

**Affiliations:** ^1^ Department of Pharmacology and Toxicology University of Vienna Vienna Austria; ^2^ Drugs Regulatory Authority of Pakistan Telecom Foundation (TF) Complex Islamabad Pakistan

**Keywords:** CaMKII, Fyn kinase, macromolecular complex, Na_V_1.5, phosphorylation, PKA, PKC

## Abstract

Cardiomyocytes are highly coordinated cells with multiple proteins organized in micro domains. Minor changes or interference in subcellular proteins can cause major disturbances in physiology. The cardiac sodium channel (Na_V_1.5) is an important determinant of correct electrical activity in cardiomyocytes which are localized at intercalated discs, T‐tubules and lateral membranes in the form of a macromolecular complex with multiple interacting protein partners. The channel is tightly regulated by post‐translational modifications for smooth conduction and propagation of action potentials. Among regulatory mechanisms, phosphorylation is an enzymatic and reversible process which modulates Na_V_1.5 channel function by attaching phosphate groups to serine, threonine or tyrosine residues. Phosphorylation of Na_V_1.5 is implicated in both normal physiological and pathological processes and is carried out by multiple kinases. In this review, we discuss and summarize recent literature about the (a) structure of Na_V_1.5 channel, (b) formation and subcellular localization of Na_V_1.5 channel macromolecular complex, (c) post‐translational phosphorylation and regulation of Na_V_1.5 channel, and (d) how these phosphorylation events of Na_V_1.5 channel alter the biophysical properties and affect the channel during disease status. We expect, by reviewing these aspects will greatly improve our understanding of Na_V_1.5 channel biology, physiology and pathology, which will also provide an insight into the mechanism of arrythmogenesis at molecular level.

## 1 INTRODUCTION

The Na_V_1.5 channel is the major isoform of the population of sodium channels in human heart responsible for the depolarizing phase of the action potential and conduction of the cardiac impulse. Na_V_1.5 is encoded by the *SCN5A* gene, located on the shorter arm of chromosome 3p21.^1^ The reported half‐life of Na_V_1.5 is within the range of 17‐35 hours,^2^, ^3^ and during its life cycle Na_V_1.5 interacts with multiple protein partners forming a macromolecular complex. These interacting partners regulate gene transcription, protein synthesis, trafficking, membrane incorporation, channel function and finally degradation. Post‐translational modifications, especially phosphorylation, play a crucial role throughout the lifecycle of Na_V_1.5 channels. Multiple kinases phosphorylate and regulate Na_V_1.5 channel physiology and pathology. Cyclic AMP‐dependent protein kinase (PKA), protein kinase C (PKC) and calcium/calmodulin‐dependent kinase II (CaMKII) are among the most abundant kinases expressed in the left ventricle of the heart, according to proteomic studies.^4^ Na_V_1.5 channel function and its regulation are in themselves complex processes, becoming ever more complex as new interacting protein partners are identified. In this review, we summarize structure and function of the Na_V_1.5 channel, formation of the macromolecular complex, its subcellular distribution and modulation by phosphorylation.

## 2 STRUCTURE AND FUNCTION

The cardiac sodium channel consists of one α‐ (Na_V_1.5) and one or more auxiliary β‐subunits in a 1:1 ratio. The Na_V_1.5 adult or canonical isoform is composed of 2016 amino acid residues with a molecular mass of about 260 kDa.^5^, ^6^, ^7^ Five different β‐subunits (β_1_‐β_4_ and β_1B_) are expressed in cardiac tissue. The β‐subunits share a common membrane topology including an extracellular N‐terminal that adopts an immunoglobulin fold, a transmembrane domain and an intracellular C‐terminal domain. The subunit β_1B_ is an exception that is a splice variant of β_1_ which lacks a transmembrane domain. The β_1_ and β_3_‐subunits associate with the Na_V_1.5 channel α‐subunit non‐covalently, while β_2_ and β_4_‐subunits are linked covalently by disulfide bonds.^5^, ^8^ These non‐pore forming β‐subunits are implicated in the physiology and pathology of the α‐subunit and play an important role in regulating the kinetics, gating, surface expression and voltage dependence of the Na_V_1.5 channel.^5^, ^9^

**Figure 1:**
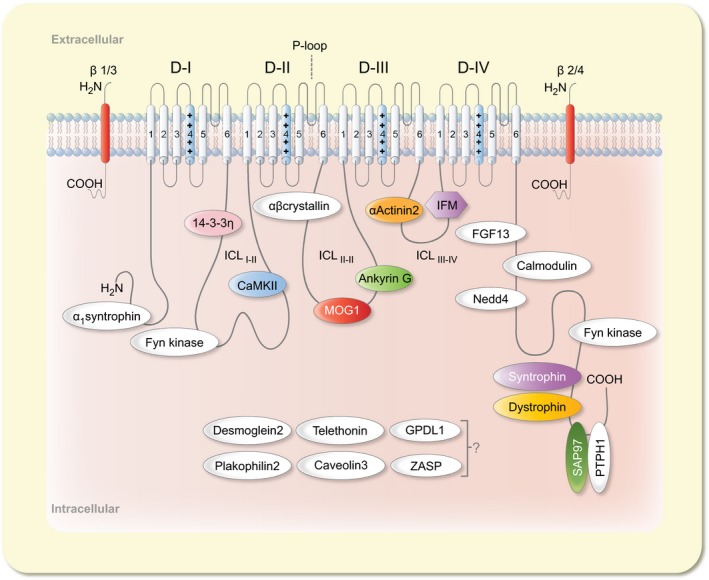
Schematic representation of cardiac sodium channel. The Na_V_1.5 α‐subunit consists of four domains (D_I_‐D_IV_), connected by intracellular loops (ICL_I__‐_ _II_‐ICL_III__‐_ _IV_). Each domain is further comprised of six transmembrane subunits (S_1_‐S_6_). The S_4_ subunit of each domain constitutes the voltage sensor while IFM motif in ICL_III__‐_ _IV_ plays a critical role in channel inactivation. The extracellular loop between S_5_ and S_6_ of each domain form P‐loop which acts as a selectivity filter. Various Na_V_1.5 protein partners are shown in different colours which correspond to the formation of macromolecular complex as shown in Figure 2. The position of these protein partners in IC‐loops, N‐ and C‐terminal is according to their approximate interaction site with the Na_V_1.5 α‐subunit

Na_V_1.5 α‐subunit RNA is a product of 28 different exons. Exon 1 and part of exon 2 encode the 5′‐untranslated region; the protein‐coding region spans exons 2‐28, while the 3′‐untranslated region is encoded by exon 28.^1^ Alternative splicing results in the production of several Na_V_1.5 RNA transcripts which can be categorized into functional (Na_V_1.5a, Na_V_1.5c, Na_V_1.5d, Na_V_1.5e and hH1c) and non‐functional (Na_V_1.5b, Na_V_1.5f and C‐terminal splice variant) splice variants.^7^, ^10^ Na_V_1.5 channel protein has a modular structure consisting of four domains (D_I_‐D_IV_), which are connected by intracellular connecting loops (ICL_I‐II_, ICL_II‐III_, and ICL_III‐IV_). In addition to intracellular connecting loops, both carboxyl terminus (C‐terminus) and amino terminus (N‐terminus) are also located intracellularly. Each domain is further comprised of six transmembrane segments (S_1_‐S_6_), which are connected by short, alternating, intra‐ and extracellular loops.^11^ The transmembrane subunit S_4_ of each domain contains positively charged amino acids at every third or fourth position and serves as a voltage sensor.^12^ The S_5_ and S_6_ subunits of each domain constitute the pore lining, and are connected by loops called P‐loops which curve back into the pore and form the selectivity filter (a group of four amino acid residues: aspartic acid, glutamic acid, lysine and alanine; DEKA arrangement). Of these four amino acids, lysine in D_III_ is vital for differentiation between monovalent Na^+^ and divalent Ca^++^ ions (Figure 1).^13^, ^14^, ^15^

Voltage‐dependent gating is a process by which alteration in membrane potential, results in structural conformations causing the ion channels to open (conductive) or close (nonconductive). Na_V_1.5 channels are activated (opened) by the outward movement of voltage sensor S_4_ and allow Na^+^ influx. Initially, Na^+^ permeability increases rapidly during phase 0 of the action potential and then decreases due to Na_V_1.5 channel inactivation (closed), which renders Na_V_1.5 refractory until repolarization is completed.^6^ The kinetics of inactivation can be subdivided into slow inactivation which develops over several seconds and regulates excitability, while fast inactivation which occurs within milliseconds is important in action potential repolarization.^16^, ^17^ The process of slow inactivation is not well understood; involvement of P‐loops and various conformational states are assumed to lead the channel into slow inactivation.^16^, ^18^ The mechanism of fast inactivation on the other hand, is well established and an inactivation gate comprising the amino acid residues isoleucine, phenylalanine and methionine (IFM), has been identified in ICL_III‐IV_. Scanning mutation analysis has identified several amino acids in the short intracellular connecting loops of transmembrane segments S_4_ and S_6_ in D_III_ and D_IV_, which serve as docking sites for inactivation and closing of the channel pore.^13^, ^15^ The C‐terminus is also known to modulate Na_V_1.5 channel inactivation by stabilizing and minimizing channel reopening.^19^ Na_V_1.5 channel activation derives its voltage dependence from outward movement of the voltage sensor, S_4_, in response to alteration in membrane potential. This outward movement of the S_4_ subunit also initiates inactivation, thus deriving its voltage dependence by coupling with the process of activation.^6^, ^20^ Na_V_1.5 channels can be activated again during phase 4 of the action potential after recovering from inactivation, although, some channels (< 1%) may reactivate during phase 2 and 3 of the action potential and generate small late sodium current (late I_Na_), also called persistent non‐inactivating current. This small inward current is usually less than 0.5% of the peak I_Na_ but it flows approximately 300‐400 milliseconds longer thereby maintaining action potential plateau and playing an important role in Na^+^ loading. Increased intracellular Na^+^ levels also increase Ca^++^ levels via the Na^+^/Ca^++^‐exchanger hence also affecting contraction and relaxation.^21^ Late I_Na_ has minimal contribution to the action potential under physiological conditions, but plays an important role in the pathological context. Late I_Na_ is increased in acquired disease conditions like heart failure (HF), hypertrophy and diabetes mellitus (DM) or under congenital cardiac disorders like long‐QT syndromes (LQTS).^21^, ^22^ This increased Late I_Na_ can trigger early afterdepolarizations by prolonging action potential duration or delayed afterdepolarizations by increasing intracellular Ca^++^ levels thus contributing to arrythmogenesis.^21^, ^23^, ^24^

Alterations in the *SCN5A* gene can lead to Na_V_1.5 channel dysfunction resulting in either gain‐of‐function or loss‐of‐function effects. These mutations in the Na_V_1.5 channel affect structure, function, trafficking, interaction with other protein partners and formation of the macromolecular complex. Na_V_1.5 channel variants have been associated with several congenital cardiac disorders such as atrial standstill, atrial fibrillation (AF), Burgada syndrome (BrS), cardiac conduction disease (CCD), dilated cardiomyopathy (DCM), LQTS and sudden infant death syndrome (SIDS).^25^, ^26^ Gain‐of‐function mutations in Na_V_1.5 result in increased persistent current which may lead to long‐QT syndrome type 3 (LQTS‐3), while loss‐of‐function mutations result in decreased peak I_Na_ and are implicated in BrS and sick sinus syndrome (SSD). The phenotype of these Na_V_1.5 channel mutations depends on several factors which may include genetic, transcriptional, translational and post‐translational modifiers. Discussion of these modifiers affecting the phenotype of disease‐causing mutant channels is beyond the scope of this review and we refer interested readers to some excellent review articles for further reading.^9^, ^26^, ^27^

## 3 MACROMOLECULAR COMPLEX AND SUBCELLULAR DISTRIBUTION

**Figure 2:**
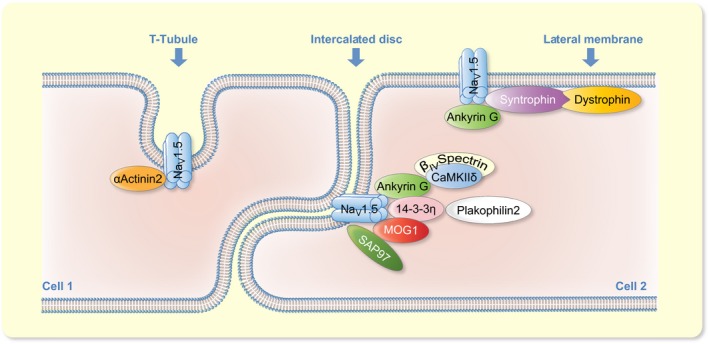
Na_V_1.5 channel macromolecular complex. Representative figure showing formation of cardiac sodium channel macromolecular complex at T‐tubules, intercalated discs and lateral membranes. The clustering of interacting protein partners at respective sites is known from the literature

Cardiac myocytes are rod‐shaped cells, approximately 100 μm in length and 20 μm in width, expressing myriad of proteins in different membrane compartments. These proteins are precisely localized indicating their distinct functional roles. Depending on subcellular localization, Na_V_1.5 channels are known to be arranged in three different compartments on plasma membrane, namely intercalated discs, lateral membranes and T‐tubules.^28^ Intercalated discs (IDs) are highly coordinated structures located between the ends of myocytes along their 20 μm breadth, and comprising both communication (gap junction) and anchoring complexes (desmosomes and adherens junctions).^29^ Mutations or acquired diseases that disrupt ID components are known to contribute in arrythmogenesis. The second and third populations of Na_V_1.5 channels reside at lateral membranes which also comprise invaginations called T‐tubules. This population of sodium channels ensures propagation of electrical impulse both in longitudinal and transverse directions (Figure 2).^28^

The Na_V_1.5 channel contains several binding motifs for auxiliary proteins which interact to regulate its intracellular transport, cellular localization, gating and degradation (Figure 1). These auxiliary proteins can be broadly categorized as adaptor proteins (anchor Na_V_1.5 channel to cytoskeleton for trafficking and targeting to specific compartments of plasma membrane), enzymes (modify Na_V_1.5 by post‐translational modifications) and regulatory proteins (modulate gating).^9^ Na_V_1.5 ICL_I‐II_ contains several sites for phosphorylation and an interaction motif for a dimeric cytosolic adaptor protein 14‐3‐3η, a 30 kDa protein widely distributed in tissues of different organisms. Seven isoforms of the 14‐3‐3 protein are expressed in mammalian cells that interact with other protein partners by two consensus sequences known as “mode‐1” (RXXpS/pTXP) and “mode‐2” (RXXXpS/pTXP) binding motifs.^30^ In cardiomyocytes 14‐3‐3η is co‐localized with Na_V_1.5 at the ID and its interaction with Na_V_1.5 shifts steady‐state inactivation towards hyperpolarization, with slowed recovery from inactivation.^31^ Recently it has been shown that 14‐3‐3η facilitates Na_V_1.5 channel α‐α dimerization and mediates coupled gating.^32^ These findings are quite intriguing given the widely held hypothesis that sodium channels exist as a single α‐subunit in macromolecular complexes.

Ankyrin‐G and a cofactor of Na_V_1.5, multi‐copy suppressor of GSP1 (MOG1), interact with ICL_II‐III_. Both ankyrin‐G and MOG1 are co‐localized with Na_V_1.5 at the ID. MOG1 is considered a cofactor for optimal expression of Na_V_1.5,^33^ while ankyrin‐G organizes trafficking of Na_V_1.5 at IDs and T‐tubules.^34^ Ankyrin‐G is known to interact via the residue sequence 1047‐VPIAVAESD‐1055 in ICL_II‐III_,^34^ and coordinates a functional macromolecular complex at IDs with Na_V_1.5, β_IV_‐spectrin and CaMKII.^35^ β_IV_‐spectrin interacts with the C‐terminal domain of CaMKIIδ and transgenic mice lacking this interaction show reduced levels of CaMKIIδ at intercalated discs, while levels in T‐tubules remain unaffected, indicating distinct trafficking mechanisms at each subcellular compartment. Recruitment of CaMKII by β_IV_‐spectrin at IDs in the vicinity of Na_V_1.5 is followed by sodium channel phosphorylation, while disruption of this interaction, as observed in *qv* ^*3J*^ transgenic mice, results in an increased I_Na_, rightward shift in fast inactivation and decreased late I_Na_.^36^ Cardio‐specific ankyrin‐G knockout mice exhibit reduced I_Na_, decreased expression and localization of Na_V_1.5 specifically at IDs of cardiomyocytes. At the same time, expression and localization of CaMKIIδ and β_IV_ spectrin to IDs was decreased resulting in disruption of late I_Na_ regulation by ankyrin‐G, β_IV_ spectrin, CaMKIIδ and the Na_V_1.5 macromolecular complex.^28^, ^35^ Ankyrin‐G also interacts with plakophilin‐2 and connexin43 (desmosomal and gap junction proteins),^37^ and is thus apparently an important player in coordinating mechanical and electrical signalling at IDs in cardiomyocytes (Figure 2).^38^ ICL_III‐IV_ is known to interact with α‐actinin_2_ which is an F‐actin cross‐linking protein. Both proteins co‐localize at T‐tubules and the interaction increases surface expression of Na_V_1.5 without any changes in channel gating.^39^, ^40^

The last three amino acids (SIV) of the Na_V_1.5 C‐terminus constitute a PDZ domain binding site which interacts with syntrophins, synapse associated protein 97 (SAP_97_) and protein tyrosine phosphatase 1 (PTPH_1_).^41^ Syntrophins form a macromolecular complex with dystrophins and coordinate localization of Na_v_1.5 at lateral membranes (Figure 2).^42^ Dystrophin knockout mice exhibit conduction defects and reduced Na_V_1.5 expression,^42^ while SAP_97_ together with ankyrin‐G, ensures correct surface expression of Na_V_1.5 at IDs.^28^, ^39^ Genetically modified Na_V_1.5 (ΔSIV) mice, in which binding of syntrophin and SAP_97_ has been disrupted, demonstrate downregulation of Na_V_1.5 channels at lateral membranes, with slowed cardiac impulse conduction.^43^ PTPH_1_ interaction affects Na_V_1.5 gating by shifting the availability curve towards hyperpolarized potentials.^44^ A calmodulin (CaM) binding IQ‐motif is also present at the C‐terminus of the Na_V_1.5 channel.^45^ CaM is a small ubiquitous 17 kDa protein which binds/senses calcium ions. Its interaction with Na_V_1.5 by binding through the IQ‐motif results in enhancing the slow inactivation process and a hyperpolarizing shift in the *I‐V* curve.^45^, ^46^ Recently, two additional interaction sites in ICL_III‐IV_ have been reported for CaM which modulates the Na_V_1.5 channel by destabilizing the inactivated state and promoting faster recovery from inactivation.^47^ The size of the Na_V_1.5 channel population is determined by a balance between synthesis and degradation. An ubiquitin protein ligase (NEDD_4‐2_) binding pY‐motif has been identified at the C‐terminus of the Na_V_1.5 channel.^48^ E_3_ ubiquitin‐protein ligase NEDD_4‐2_ ubiquitinates the Na_V_1.5 channel, thus giving the signal for internalization and finally degradation.^48^ Co‐expression of NEDD_4‐2_ with Na_V_1.5 in *Xenopus* oocytes, decreased ionic currents by up to 40‐65% with significant reduction in membrane expression. Conversely, an inactive NEDD_4‐2_ analogue increased both I_Na_ and Na_V_1.5 membrane expression.^48^, ^49^ Several modulators such as 14‐3‐3 protein, MAPKs, PKA and serine/threonine kinase SGK regulate NEDD_4_.^27^, ^50^ Recently, αB‐crystallin was reported to interact with Na_V_1.5 via ICL_II‐III_ and C‐terminus. αB‐crystallin co‐localizes with Na_V_1.5 and increases I_Na_ by decreasing the ubiquitination.^51^ Additionally, fibroblast growth factor homologous factors like FGF_12_, FGF_13_ and FGF_14_ also interact with the C‐terminus of Na_V_1.5 affecting the expression, trafficking and gating of the channel.^52^, ^53^

Lastly, the role of the N‐terminus of Na_V_1.5 is not fully understood yet; however, certain missense mutations in the N‐terminus lead to degradation of the channel and exert a negative effect on wild‐type (WT) channels. Also co‐expression of the N‐terminus peptide fragment with WT channels resulted in a twofold increase in surface expression and I_Na_ compared to WT channel alone. This indicates that α‐subunits of Na_v_1.5 channel interact via the N‐terminus by unknown mechanisms.^54^ Recently, it has been shown that an internal PDZ‐like domain, present in the N‐terminus of Na_V_1.5 interacts with α_1_‐syntrophin, exerting a chaperone‐like effect to positively modulate Na_V_1.5, Kir2.1 and Kir2.2 channels in cardiomyocytes.^55^ Moreover, several phosphorylation sites for Fyn kinase have also been identified in the N‐terminus which may contribute to a depolarizing shift of fast inactivation, produced by Fyn kinase interaction with Na_V_1.5 channel.^56^ Caveolin‐3, desmoglein‐2, glycerophosphoryl diester phosphodiesterase‐like protein 1 (GPDL_1_), plakophilin‐2, telethonin and Z‐band alternatively spliced PDZ‐motif protein (ZASP), are also reported to interact with Na_V_1.5 channel through unidentified sites (Figure 1).^39^ The specific purpose of these distinct pools is not well established, but based on recent studies the proportions of current generated by these pools can be speculated. Sodium channels present on T‐tubules generate around 20%, while Na_V_1.5 channels residing on lateral membranes about 30% of I_Na_. Thus, the remaining 50% of I_Na_ may be attributed to Na_V_1.5 channels localized on IDs.^28^

## 4 MODULATION OF KINETICS AND TRAFFICKING OF NA_V_1.5 CHANNEL BY PHOSPHORYLATION

### 4.1 PKA

**Figure 3:**
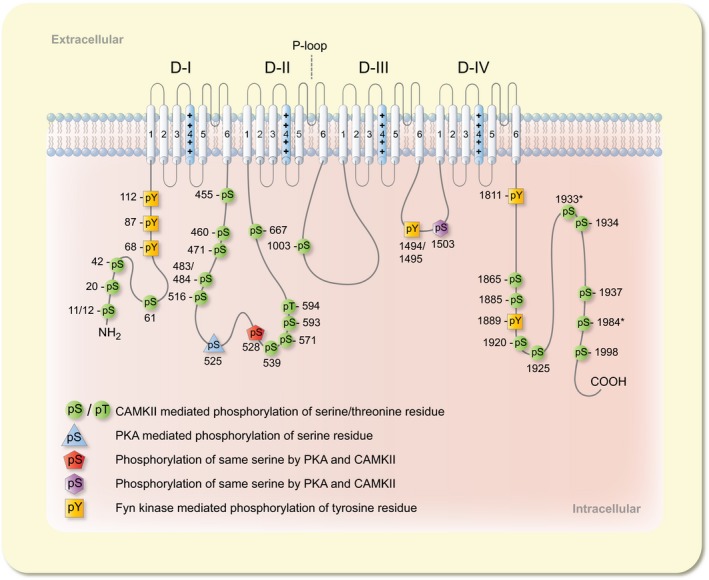
Phosphorylation of Na_V_1.5 channel. Diagrammatic presentation of phosphorylation of the Na_V_1.5 channel α‐subunit at serine, threonine and tyrosine residues by PKA, PKC, CaMKII and Fyn kinase with respective colours. The amino acids numbering is according to Na_V_1.5c isoform (Uniprot identifier # Q14524‐1), while the amino acids with asterisk signs^113^ correspond to Na_V_1.5 hH1c isoform numbering

PKA, first described in 1968,^57^ is a well‐studied protein kinase. It is a holoenzyme existing as a heterotetramer with two catalytic and two regulatory subunits. When a second messenger cAMP binds to these regulatory subunits, a conformational change takes place which releases and activates the catalytic subunits.^58^ PKA is targeted to different sub‐cellular locations by a scaffolding protein called A‐kinase anchoring protein (AKAP), where upon activation, it phosphorylates the target proteins by transferring γ‐phosphate of ATP.^59^ PKA phosphorylates myriad of proteins including the Na_V_1.5 channel.^60^ The generalized consensus motifs in substrate proteins for phosphorylation by PKA include R/KXS/T, RRXS/T and R/KXXS/T.^61^ Initial evidence for PKA mediated modulation of the Na_V_1.5 channel was observed by stimulation of the β‐adrenergic (β‐AR) system with isoproterenol which decreased I_Na_ and produced a hyperpolarizing shift in steady state inactivation by increasing levels of cAMP.^62^, ^63^ Both decreased upstroke velocity of action potential and decreased I_Na_ has been reported in neonatal rat and adult guinea pig ventricular myocytes through PKA and G‐protein‐regulated pathways.^62^, ^63^, ^64^, ^65^ Contrarily, increased I_Na_ has also been reported in guinea pig myocytes,^66^ rabbit myocytes^67^ and Na_V_1.5 expressed in *Xenopus* oocytes.^68^ This increase in I_Na_ was attributed either to phosphorylation of Na_V_1.5 by PKA or activation of G‐protein (Gsα), which was observed at hyperpolarized potentials (negative to ‐75 mV) without any effect on Na_V_1.5 channel gating kinetics.^67^ Some possible explanations for these initial conflicting reports include different voltage protocols used (depolarized vs hyperpolarized holding potentials),^62^ or different concentrations of isoproterenol used in different experimental settings.^69^, ^70^ This discrepancy in I_Na_ by cAMP or β‐AR stimulation was further elaborated in experiments showing a test pulse of ‐50 mV at a holding potential of ‐150 mV increased I_Na_, while a test pulse of +30 mV at a holding potential of ‐90 mV decreased I_Na_. However, no effect was observed when sodium currents were elicited by a test pulse of +30 mV at a holding potential of ‐150 mV.^71^ Later, with the help of biochemical studies, it was demonstrated that PKA phosphorylates serine residues at positions 526 and 529 in ICL_I‐II_ of the rat Na_V_1.5 channel. However, no functional data for the biophysical characteristics of Na_V_1.5 channel for these phosphorylated sites were provided by the authors.^72^ The involvement of ICL_I‐II_ in modulation by PKA, was also confirmed in human Na_V_1.5 channel (hH1 variant), where injection of cAMP in *Xenopus* oocytes expressing Na_V_1.5 channel increased the conductance of channel without affecting half maximal activation or inactivation.^73^ The increase in I_Na_ by PKA stimulation develops slowly without reaching saturation over a time period of one hour, suggesting the involvement of an additional mechanism other than direct modulation of the channel by phosphorylation.^74^ Whole cell patch clamp experiments in rat myocytes demonstrated that the increased I_Na_ observed upon β‐AR stimulation was due to the increased number of functional sodium channels, rather than activation by PKA and Gsα, which does not affect gating or open probability of the channel.^75^ Further evidence for the involvement of PKA in Na_V_1.5 channel trafficking was obtained by incubating Na_V_1.5 channel expressing cells with chloroquine and monensin. Both the drugs interfere in recycling of membrane proteins and pre‐incubation with these two drugs does not increase I_Na_ on PKA stimulation.^74^ These reports clearly indicate that PKA mediated phosphorylation increases I_Na_ by promoting forward trafficking and increasing total number of functional Na_V_1.5 channels.^76^, ^77^ In the rat Na_V_1.5 channel, the involvement of two serine residues at positions 526 and 529 was reported earlier to increase I_Na_ upon PKA activation.^72^ Mutation of these conserved serine residues at positions 525 and 528 to alanine (Figure 3; Table 1), in human Na_V_1.5 channel (hH1 variant), abolished the PKA‐mediated increase in I_Na_ suggesting the same mechanism may also play a role in modulation of other Na_V_1.5 channel isoforms.^78^ In ICL_I‐II_ there are three endoplasmic reticulum (ER) retention signals (RXR), R_479_KR_481_, R_533_RR_535_ and R_659_QR_661_ which are either up‐ or downstream of the PKA‐phosphorylated serine residues. Addition of a phosphate group on serine 525 or 528 imparts large negative charge on these residues which may mask the ER retention signals, thus promoting forward trafficking of the Na_V_1.5 channel. ER retention signals play an important role in trafficking of membrane proteins, since newly formed proteins are retained in the ER and released when these ER signals are masked by binding of another protein.^78^ Among the three ER‐retention sites, the R_533_RR_535_ site plays a major role in PKA‐mediated increased I_Na_; it could be argued, that phosphorylation of serine 525 and 528 could mask this site and promote forward trafficking of the Na_V_1.5 channel.^78^ This forward trafficking redistributes Na_V_1.5 channels from intracellular reservoirs like the ER and caveolae in the plasma membrane, thus increasing Na_V_1.5 channel recruitment up to 45%.^77^

**Table 1:** Modulation and phosphorylation of Na_V_1.5 channel. Phosphorylation of Na_V_1.5 channel serine, threonine and tyrosine residues by PKA, PKC, CaMKII, Fyn and SGK with the method of identification and the most commonly reported effects on Na_V_1.5 channel by the respective kinases

Kinase	Phosphorylated residue	Effect on Na_V_1.5 channel	Method of indentification	Reference
PKA	S_526_ and S_529_ in rat Na_V_1.5 S_525_ and S_528_ in human Na_V_1.5	Increased I_Na_ Hyperpolarizing shift in inactivation curve Increased forward trafficking of Na_V_1.5 channel	In vitro analysis Mutagenesis Electrophysiology 2D‐phosphopeptide analysis SDS‐PAGE and immunoblotting	62, 63, 66, 67, 68, 72, 77, 78
PKC	S_1505_ in rat Na_V_1.5 S_1503_ in human Na_V_1.5	Decreased I_Na_ Decreased single channel conductance Hyperpolarizing shift in inactivation curve Increased internalization of Na_V_1.5 channel	In vitro analysis Mutagenesis Electrophysiology	87, 88, 89, 91, 92, 93, 94, 95, 96
CaMKII	S_571_, S_1937/1938_, S_1989_ in mouse Na_V_1.5 S_11_, S_12_, S_20_, S_42_, S_61_, T_455_, S_460_, S_471_, S_483/S484_, S_516_, S_528_, S_539_, S_571_, S_593_/T_594_, S_667_, S_1003_, S_1503_, S_1865_, S_1885_, S_1920_, S_1925_, S_1934_, S_1937_, S_1998_ in human Na_V_1.5	Hyperpolarizing shift in inactivation curve Increased late I_Na_ Increased intermediate inactivation Slowed inactivation Slowed recovery from inactivation	In vitro/In situ analysis Mutagenesis Immunopurification Western blotting Mass spectrometry	36, 103, 106, 112, 113, 114, 115
Fyn	Y_68_, Y_87_, Y_112_, Y_1494/Y1495_, Y_1811_, Y_1889_ in human Na_V_1.5	Depolarizing shift in inactivation curve Accelerated recovery from inactivation Decreased intermediate inactivation	In vitro analysis Mutagenesis Immunopurification Western blotting	56, 130, 131
PI3Kα and PKB/Act	N/A	Decreased I_Na_ Increased late I_Na_ Prolonged QT interval	In vitro analysis Mutagenesis Electrophysiology	133, 134
PDK1	N/A	Decreased I_Na_ Reduced surface expression of Na_V_1.5 lower heart rate Prolonged QRS and QTc interval	In vitro analysis Mutagenesis Western blotting Electrophysiology	136
SGK	S_483_, S_664_, T_1590_ in Na_V_1.5	Increased I_Na_ Increased surface expression of Na_V_1.5 Depolarizing shift in inactivation Hyperpolarizing shift in activation curve	In vitro analysis Mutagenesis Electrophysiology	138, 139, 140
β1‐AR stimulation	S_484_, S_667_, S_670_ in mouse Na_V_1.5	N/A	In situ analysis Phosphopeptide enrichment Mass spectrometry	141
Unknown kinase	T_17_, S_457_, S_464_, S_499_, S_664_, in human Na_V_1.5	N/A	In situ analysis Immunopurification Mass spectrometry	115
Native phosphorylated residues	S_36/39/42/T38_, S_457_, S_460_, S_483_, S_484_, T_486_, S_497_, S_499_, S_510_, S_516_, S_524/525_, S_539_, S_571_, S_664_, S_667_, S_1012_, S_1888_, S_1937_, S_1938_, S_1989_ in mouse Na_V_1.5 S_42_, S_460_, S_483_, S_484_, S_497_, S_510_, S_516_, T_570_, S_571_, S_577_, S_1937_, S_2007_ in human Na_V_1.5	N/A	In situ analysis Immunopurification Mass spectrometry	113, 114, 115

PKA is a downstream effector of the β‐AR signalling pathway and plays an important role in cardiac excitation‐contraction coupling.^79^ In cardiomyocytes, β‐AR couples with stimulatory Gs protein and stimulation of the adrenergic system produces cAMP which activates PKA. β‐AR stimulation modulates Na_V_1.5 channel directly by PKA‐mediated phosphorylation or indirectly by Gs_α_ signalling pathway.^75^ PKA activation can fractionally increase I_Na_ in epicardial border zone cardiomyocytes of infarcted canine heart.^80^ Moreover, a missense mutation (R_526_H) in ICL_I‐II_ of Na_V_1.5 channel resulted in a BrS phenotype with markedly reduced I_Na_. This mutation resides in the PKA recognition site which inhibits PKA‐mediated phosphorylation of the Na_V_1.5 channel, resulting in reduced incorporation of functional channel into the plasma membrane. Activation of PKA does not increase the I_Na_ by rescuing R_526_H mutant channels, which also underpins the importance of PKA‐mediated phosphorylation of the channel.^81^ Apart from the Na_V_1.5 channel, PKA also phosphorylates Ca_V_1.2, phospholamban, troponin I and C, ryanodine receptors and several other proteins, and so plays important role in cardiac physiology.^82^ However the direct involvement of PKA in cardiac pathophysiology or the use of PKA to improve cardiac pathology is not well described; further studies are required to characterize the PKA‐Na_V_1.5 interaction as a potential target for drug discovery and to reveal its potential role in cardiac pathophysiology.

### 4.2 PKC

PKC consists of a single polypeptide with a regulatory N‐terminus and catalytic C‐terminus. This family of kinases transduces a diverse range of signals and since the identification of the first member in the 1980s, 11 different isozymes have been identified and categorized into three classes: calcium‐dependent conventional PKCs (α, β_I_, β_II_ and γ), calcium‐independent novel PKCs (δ, ε, η, θ and μ) and atypical PKCs (ζ and λ).^83^ PKC is a downstream effector of several circulating hormones such as angiotensin II (Ang‐II), endothelin and norepinephrine, which upon stimulation cause phosphorylation of several cardiac proteins, activate other kinases and alter gene expression. These processes influence impulse conduction and EC‐coupling implicated both in normal physiology and pathological conditions.^84^ Like PKA, PKC phosphorylates serine and threonine residues in substrate proteins, but compared to PKA it displays less specificity.^85^ Initially it was reported that PKC activators like TPA (phorbol ester) and 1,2‐dioctanoglycerol (diacylglycerol analogue) increased single channel sodium currents and rate of current decay in neonatal rat ventricular myocytes.^86^ Later it was shown that activation of PKC by OAG (1‐pleoyl‐2‐acetyl‐sn‐glycerol) decreases I_Na_ and creates a hyperpolarizing shift in steady‐state inactivation both in neonatal rat ventricular myocytes and rat cardiac sodium channel (rNa_V_1.5), stably expressed in the Chinese hamster lung 1610 cell line. This decrease in I_Na_ was attributed to a decreased open probability in single‐channel studies.^87^ Subsequently, the same authors reported that serine 1505 in ICL_III‐IV_ was involved in modulation of rNa_V_1.5 by PKC; upon replacement of this serine by alanine, PKC activation did not decrease I_Na_ nor did it mediate a hyperpolarizing shift in fast inactivation.^88^ This serine residue is conserved in several sodium channel isoforms and plays a role in modulation by PKC; however, the authors did not provide any direct evidence for phosphorylation of serine 1505. Similarly, in *Xenopus* oocytes transiently expressing human Na_V_1.5 (hH1 variant), activation of PKC by PMA (phorbol 12‐myristate 13‐actate) or OAG decreased I_Na_ without any hyperpolarizing shift in steady‐state inactivation. The decrease in I_Na_ was in part attributed to phosphorylation of serine 1503 (homologous to serine 1505 in rNa_V_1.5) in ICL_III‐IV_ because mutation of this serine did not completely abolish the effect of PKC activation (Figure 3; Table 1).^89^ PKC activation by phorbol esters or diacylglycerol derivatives exhibit diverse effects on cardiomyocytes. These agonists are nonspecific and have multiple targets besides PKC which may also exert nonspecific effects on Na_V_1.5 channel. To investigate these differential effects a peptide PKCP was used to specifically activate endogenous PKC and observe effects on the Na_V_1.5 channel in rat ventricular myocytes. This peptide blocks the auto‐regulatory region of native PKC and exposes the catalytic site to bring about phosphorylation of substrate proteins. Activation of PKC by PKCP peptide caused a dose dependent depolarizing shift in half maximal inactivation of the Na_V_1.5 channel, while no effect was observed on half maximal activation or peak I_Na_. This depolarizing shift in inactivation was reversed by PKC inhibitors such as chelerythrine chloride or saturosporine. Besides the depolarizing shift in the inactivation curve, PKC activation also slows I_Na_ decay and channel inactivation, while recovery from inactivation is enhanced.^90^

Human cardiomyocytes express nine different isoforms of PKCs irrespective of normal or diseased states; however, levels of these isoforms may vary in different conditions.^84^ PKC activators such as phorbol ester or diacylglycerol analogues are nonspecific in nature and it is highly likely that different PKC isoforms act differently providing a reason for differential effects reported in various studies of activation of PKC.^86^, ^87^, ^90^ By using peptide activators or inhibitors of PKC isoforms it was described that specific activation of εPKC decreases I_Na_ in rat ventricular myocytes and the human Na_V_1.5 channel (hH_1_ isoform) heterologously expressed in *Xenopus* oocytes, without affecting voltage dependence of activation or inactivation. However the blockade of εPKC did not completely abolish the effect of PMA which suggests the involvement of other PKC isoforms as evidenced in another study where activation of PKCα decreased the I_Na_.^91^, ^92^ With the development of specific PKC activators it was described that activation of PKCα decreased I_Na_ without any effect on gating. This decrease in I_Na_ was slow in development and non‐saturable which was supposedly because of internalization or re‐distribution of functional hNa_V_1.5 channels away from the plasma membrane.^92^, ^93^ This modified intracellular trafficking of hNa_V_1.5 channels was attributed to PKC‐mediated phosphorylation of the channel and ROS.^93^ Moreover, the authors also described that in pre‐blocked cPKCs, low concentrations of PKC activators such as PMA (1 nM) and thymeleatoxin (50 nM) cross activate PKA which slightly increases I_Na_ and explains initial conflicting results on PKC activation.^92^ Renin‐angiotensin signalling is known to activate several PKC isozymes and in several cardiovascular disorders this pathway is chronically activated. In transgenic mice overexpressing cardio‐specific angiotensin‐II type 1 receptors (AT1R), QRS complex widening and slower action potential was observed because of decreased I_Na_. This AngII‐AT1R pathway stimulation activates PKCα and co‐localizes it with Na_V_1.5 at the plasma membrane where their interaction results in decreased I_Na_.^94^

Recently a metabolic pathway for PKC activation has been described where the elevated levels of NADH activate PKCδ, resulting in a decrease in I_Na_.^95^ Interestingly, this decrease in I_Na_ was not because of any change in surface expression but rather channel conductance was decreased directly by phosphorylation of serine 1503 in Na_V_1.5 channels and indirectly by increasing ROS production in mitochondria.^95^, ^96^ The PKCδ antagonism completely reversed both decrease in I_Na_ and ROS production from the mitochondria while specific inhibition of PKCα could partially recover I_Na_ without any effect on ROS production, thus indicating the involvement of more than one PKC isozyme. By mutational analysis it was described that both phosphorylation of serine 1503 in Na_V_1.5 channels and ROS production in mitochondria are required for PKCδ‐mediated modulation of the Na_V_1.5 channel.^95^ Alteration in sodium current is detrimental in underlying disease conditions and may trigger arrhythmias leading to sudden cardiac death.^94^ As discussed above, activation of several PKC isozymes are reported to influence the I_Na_ and the reported effects on Na_V_1.5 are contradictory, which might be because of involvement of different PKC isozymes. Activation of PKC results in phosphorylation of a conserved serine residue in ICL_III‐IV_ which alters Na_V_1.5 channel trafficking, but besides this serine residue phosphorylation of other serine residues is also likely. Additional detailed studies are warranted which might unravel further aspects of PKC signalling and may explain the differential effects reported on Na_V_1.5 channel by PKC activation.

### 4.3 CaMKII

CaMKII is a multifunctional enzyme class consisting of four isoforms. The α‐ and β‐ isoforms are mainly expressed in neuronal tissues while γ‐ and δ‐ are expressed ubiquitously.^97^ Alternative splicing of primary transcripts results in generation of multiple variants. Six splice variants of the CaMKIIδ isoform have been identified in the heart.^98^ As the name indicates, CaMKII activity is dependent on Ca^2+^ and/or CaM, which after binding induces conformational changes in CaMKII to autophosphorylate at threonine 287. Autophosphorylation keeps CaMKII active after dissociation of Ca^2+^/CaM.^99^ CaMKII is also activated in a Ca^2+^ independent manner by ROS‐mediated oxidation of methionine residues at positions 281/282. This mode of activation requires initial binding of Ca^2+^/CaM to expose the potential methionine residues followed by oxidation. Oxidative stress is observed in HF, after myocardial infarction (MI) and with increased levels of Ang‐II.^100^ Moreover levels of CaMKIIδ_C_ are also increased during pressure overload, HF and sustained β_1_‐AR stimulation thus both factors contributing in outcomes of adverse cardiac events.^101^, ^102^, ^103^ Hence, CaMKII Inhibition appears to be cardio‐protective after MI by reducing apoptosis and remodelling associated with excessive stimulation of β‐AR signalling and Ang‐II.^100^, ^104^

Initial evidence for CaMK‐mediated modulation of Na_V_1.5 channel was obtained indirectly using CaMK inhibitors such as KN‐93 and autocamtide‐2 related inhibitory peptide (AIP). KN‐93 slowed Na_V_1.5 channel current decay, produced a depolarizing shift in fast inactivation and slowed entry into inactivated states. Interestingly, AIP the specific inhibitor of CaMKII did not exhibit any effects on Na_V_1.5 channels and the authors suggested the involvement of CaMKIV in modulation of Na_V_1.5 channels.^105^ Direct evidence for CAMKII interaction with Na_V_1.5 channel was obtained from transgenic mice expressing CaMKIIδ_C_ and in rabbit ventricular myocytes with acute overexpression of CaMKIIδ_C_. Overexpression of CaMKIIδ_C_ increased the phosphorylation of Na_V_1.5 channels and in both experimental models CaMKIIδ_C_ interaction slowed Na_V_1.5 channel fast inactivation, created a hyperpolarizing shift in steady‐state inactivation, increased late I_Na_ and number of Na_V_1.5 channels undergoing intermediate inactivation along with slowed recovery from inactivation. Together these effects could be arrhythmogenic (prolonged QT and QRS intervals) as has been observed in transgenic mice overexpressing CaMKIIδ_C_.^106^ CaMKII inhibition by KN‐93 reduced late I_Na_ in ventricular myocytes from normal and experimental canine model of chronic HF.^107^ CaMKII inhibition by KN‐93 under basal physiological conditions in rat ventricular myocytes reduced peak I_Na_ and late I_Na_, produced a hyperpolarizing shift in fast inactivation, and augmented intermediate inactivation with slowed recovery from both fast and slow inactivation. It also reduced membrane excitability by decreasing upstroke velocity during action potential.^108^ These results are not consistent with overexpression of CaMKII,^106^ which caused a hyperpolarizing shift in inactivation and slowed recovery from inactivation. In guinea pig ventricular myocytes CaMKIIα increased peak I_Na_, produced a depolarizing shift in fast inactivation, accelerated recovery from inactivation, increased late I_Na_ and decreased the fraction of channels undergoing intermediate inactivation. CaMKIIα also increased action potential duration while blockade by KN‐93 shortened it. Moreover, CaMKIIα‐mediated phosphorylation was observed in the ICL_I‐II_ and C‐terminal domains of the Na_V_1.5 channel.^46^ These results differ in certain aspects from rabbit ventricular myocytes overexpressing CaMKIIδ_C_, which demonstrated hyperpolarizing shift in fast inactivation, slowed recovery from inactivation and increased intermediate inactivation.^106^ These differences can be accounted for by different methodological approaches, different CaMKII (α vs δ) isoforms, altered phosphorylation of Na_V_1.5 channel due to overexpression of CaMKIIδ_C_ and association of the chronic overexpression of CaMKIIδ_C_ with cellular and structural remodelling. The effects of CaMKII on Na_V_1.5 remained disputed; however, there is a general consensus on increased late I_Na_, but sodium channel gating and kinetics are debatable. Late I_Na_ is increased in certain pathological conditions like LQTS‐3, cardiac ischemia or HF. Increased late I_Na_ overloads cellular calcium and sodium levels, which may play a key role in diastolic dysfunction and arrythmogenesis. Late I_Na_ prolongs action potential duration by reducing repolarization reserves and may trigger early after depolarizations, while sodium and calcium overload may lead to delayed after depolarizations both in atrial and ventricular myocytes.^109^ Pathological or drug‐induced, increased late I_Na_ elevates Na^+^ levels in cardiomyocytes, which in turn elevates Ca^+2^ levels by sodium calcium exchanger thus paradoxically activating CaMKII.^21^, ^23^, ^24^, ^110^ Inhibition of late I_Na_ by ranolazine prevents CaMKII activation and acts as a cardio‐protective agent.^111^

CaMKII interaction with Na_V_1.5 in myocytes has been established by co‐immunoprecipitation experiments and immunostaining which indicates co‐localization of CaMKII and Na_V_1.5 channels at IDs and T‐tubules.^36^, ^106^, ^108^ These co‐immunoprecipitation experiments and co‐localization of CaMKII with Na_V_1.5 channels strongly suggest direct interaction of CaMKII with the Na_V_1.5 channel. There are several CaMKII‐mediated phosphorylation motifs (RXXS/T) in Na_V_1.5 channel and alanine‐scanning suggested phosphorylation of serine residue at position 571 which was also validated by site‐specific antibodies. In transgenic *qv* ^*3J*^ mice, decreased phosphorylation of serine 571 was observed following disruption of interaction of CaMKII with the Na_V_1.5 channel.^36^ Activated CaMKIIδ_C_ interacted stably with ICL_I‐II_ of Na_V_1.5, the loop which has been shown to contain more than one phospho‐acceptor sites for CaMKII. In vitro phosphorylation of Na_V_1.5 by CaMKIIδ_C_ showed phosphorylation of serine 483/484, 516 and serine 593/threonine 594, but the authors^112^ detected no phosphorylation on serine 571^35^, ^36^ as previously reported. Further biochemical tests established that only phosphorylation on serine 516, 593 and threonine 594 are involved in CaMKIIδ_C_‐mediated modulation of Na_V_1.5 with preference order of 516 > 594 > 593. Alanine mutagenesis of serine 516, 571 and threonine 594, but not serine 593, prevented a CaMKIIδ_C_ mediated hyperpolarizing shift in sodium channel availability and intermediate inactivation, indicating their phosphorylation‐dependent role in regulation of Na_V_1.5 by CaMKIIδ_C_.^112^ Mass‐spectrometry based analysis of in situ immunopurified Na_V_1.5 channel revealed eleven phosphorylated residues, of which 10 reside in ICL_I‐II_ (serine 457, 460, 483, 484, 497, 510, 524/525, 571, 664, 667), and one resides in the N‐terminus (serine 36/39/42/threonine38). Several interacting protein partners, including CaMKII ‐β, ‐δ, ‐γ subunits, CAM, and FGF‐13 were also identified by mass‐spectrometry based analysis from immunoprecipitated Na_V_1.5 of mouse ventricular myocytes by the same authors.^113^ Recently, nine more Na_V_1.5 phosphorylated serine and threonine residues (486, 499, 516, 539, 1012, 1888, 1937, 1938 and 1989) have been reported by the same research group investigating WT and transgenic mice overexpressing CaMKIIδ_C_. Among these, phosphorylation of serine residues at position 1938 and 1989 was increased by CaMKIIδ_C_ overexpression. Both serine residues (1938 and 1989) are conserved in the human Na_V_1.5 channel and when the orthologous serine residues at position 1933 and 1984 in human Na_V_1.5 (hH1c) are mutated, interaction of FGF‐13 with Na_V_1.5 channels is disrupted; this alters fast inactivation, increases late I_Na_ and decreases channel availability.^114^ In another recent study, mass‐spectrometry based analysis of immunopurified and CaMKIIδ_C_‐mediated in vitro phosphorylation of human Na_V_1.5 expressed in HEK‐293 cells revealed 31 serine and three threonine phosphorylated residues.^115^ Fifty percent of these phosphorylation sites were located in ICL_I‐II_, underpinning the importance of the first IC‐loop in modulation of Na_V_1.5 by post‐translational modifications. Of these phosphorylated residues 17 were present at baseline while 23 residues were phosphorylated by CaMKIIδ_C_. Phosphorylated amino acids were more scattered in the ICL_I‐II_, while they were clustered together in the N‐ and C‐termini.^115^ At the N‐terminus, serine 11, 12, 20, 42 and 61 were phosphorylated by CaMKIIδ_C_, and of these, serine 42 was also phosphorylated at baseline while threonine 17 was phosphorylated by an unknown kinase. Eighteen phosphorylated residues were present in ICL_I‐II_ and seven of them (serine 460, 471, 484, 516, 528, 539, 571, 667 and threonine 455) were phosphorylated by CaMKIIδ_C_. Serine 460, 483, 484, 497, 510, 516, 577 and threonine 570 were also phosphorylated at baseline, while serine 457, 464, 499 and 664 were phosphorylated by an unknown kinase. ICL_II‐III_ and ICL_III‐IV_ contained one phosphorylated residue each, with serine 1003 and 1503, respectively. Eight phosphorylated residues were identified in the C‐terminus and seven of them (serine 1865, 1885, 1920, 1925, 1934, 1937 and 1998) were phosphorylated by CaMKIIδ_C_. Two serine residues 1937 and 2007 were also phosphorylated at baseline (Figure 3; Table 1).^115^

The majority of these identified phosphorylation sites are not characterized functionally; however, several are well described in pathological processes. Secondly, it may be emphasized that the phosphorylation status can differ on the same residue under normal physiological or pathological conditions and the same residue can be phosphorylated by multiple kinases.^113^ Phosphorylation at S516 is decreased in HF patients and it is also known that methylation at arginine 513 decreases phosphorylation of serine at position 516 indicating intriguing crosstalk between methylation and phosphorylation.^115^, ^116^, ^117^ CaMKII‐mediated phosphorylation of serine 571 specifically regulates the late I_Na_ component of Na_V_1.5 without affecting channel availability and recovery from inactivation. This serine 571 mediated increased late I_Na_ prolongs APD and QT interval with increased susceptibility to arrhythmias. Ventricular myocytes from transgenic mice with hyperphosphorylation at position 571 show increased late I_Na_ and β‐AR stress induced afterdepolarizations with repolarization abnormalities. Moreover, phosphorylation at S571 contributes in HF, but not in pressure overload hypertrophy.^118^ Two proarrhythmic Na_V_1.5 variants (A_572_D and Q_573_E) were initially identified in extensive genetic analysis of LQTS probands.^119^, ^120^, ^121^ Both of these variants reside near the CaMKII phosphorylation site serine 571. Although these variants do not have any effect on serine 571, this substitution introduces negative charge which simulates a phosphorylated residue. Both these variants exhibited increased late I_Na_ which was reversed by ranolazine and produced hyperpolarizing shift in fast inactivation with slowed recovery from inactivation. Lengthening of action potential duration was also observed with afterdepolarizations, which were eliminated by ranolazine.^122^ Effects produced by these variants recapitulate CaMKII mediated effects, and computational models indicate that both variants show structural and electrostatic features similar to the serine 571 phosphorylated channel. Phosphorylated CaMKII and CaMKII mediated increased phosphorylation of serine 571 was observed in ventricular myocytes of non‐ischaemic HF patients and in canine post infarct border zone, but not in transgenic mice AC3‐I expressing CaMKII inhibitor.^122^ CaMKII‐mediated Na_V_1.5 channel phosphorylation and regulation exhibits both gain‐of‐function (increased late I_Na_) and loss‐of‐function (decreased availability) effects, indicating complex regulation by phosphorylation. Moreover, further detailed studies are required to link CaMKII‐mediated phosphorylated residues with pathological process of cardiac disorders and their role in disease progression.

### 4.4 Fyn kinase

Besides phosphorylation of serine and threonine, the Na_V_1.5 channel is also modulated by phosphorylation of tyrosine residues. Initial evidence for tyrosine phosphorylation of the Na_V_1.5 channel was reported indirectly by the use of protein tyrosine kinase inhibitors (genistein, AG957, PP2 and ST638), which all decreased I_Na_ while genistein and AG657 also produced a hyperpolarizing shift in steady‐state fast inactivation and prolonged recovery from inactivation.^123^ Similarly, co‐expression of PTPH1 with Na_V_1.5 also shifts steady‐state inactivation towards hyperpolarized potentials.^44^

Fyn tyrosine kinase is a member of the non‐receptor Src family of tyrosine kinases which is expressed ubiquitously.^124^ Fyn kinase consists of four domains (SH_1_ ‐ SH_4_). The SH_1_ domain possesses tyrosine kinase activity and is located in the C‐terminal domain. The SH_2_ domain recognizes phosphorylated tyrosine while the SH_3_ domain is non‐catalytic and binds target proteins through proline‐rich regions.^124^ The generalized proline‐rich recognition motifs of the SH_3_ domain are categorized as class I or class II and have the sequence R/KXXPXXPX and PXXPXR/K, respectively.^125^, ^126^ The SH_4_ domain constitutes a palmitoylation and myristoylation sequence for membrane anchoring and is located at the N‐terminus.^124^ In cardiomyocytes Fyn kinase regulates stability of adherens junctions and is also localized at caveolae along with Na_V_1.5 channels.^127^, ^128^, ^129^ Interaction of Fyn kinase with Na_V_1.5 has been established where it co‐immunoprecipitates and phosphorylates the Na_V_1.5 channel.^130^ This phosphorylation creates a depolarizing shift in steady‐state fast inactivation, increases recovery from inactivation and decreases rate of entry into slow inactivation.^131^ Interaction of Fyn kinase with Na_V_1.5 is complex and involves multiple steps. Binding of Fyn kinase to proline‐rich regions in the ICL_I‐II_ domain and C‐terminus is followed by phosphorylation of nearby tyrosine residues in the N‐terminus (Y_68_, Y_87_, and Y_112_), ICL_III‐IV_ (Y_1494_, Y_1495_)^130^, ^131^ domain and C‐terminus (Y_1811_, Y_1889_), indicating a complex and multistep modulation.^56^

### 4.5 Phosphoinositide 3‐kinase signalling

Phosphoinositide 3‐kinases (PI3K) are a group of kinases that phosphorylate the 3‐hydroxyl group of inositol in phosphoinositides. They are categorized into three classes (class‐1, class‐2 and class‐3) depending upon structure, subunits and substrate specificity. The mammalian heart expresses different isoforms from the three classes of PI3Ks but most of the studies have focused on describing the role of class‐1 PI3Ks in cardiac electrophysiology. Class‐1 PI3Ks are heterodimers consisting of a catalytic subunit (PI3Kα, PI3Kβ, PI3Kγ or PI3Kδ) bound to a regulatory subunit.^132^ Specific inhibition of PI3Kα in mouse ventricular myocytes increased action potential duration by prolonging the QT interval, decreasing peak I_Na_ and increasing the late I_Na_, while no effect was observed on the blockade of other three catalytic subunits.^133^, ^134^ The downstream effectors of PI3K pathway are protein kinase Akt/PKB and 3‐phosphoinositide dependent protein kinase 1 (PDK1). Downregulation of Akt is observed in diabetes as PI3K signalling is reduced due to decreased insulin levels which increase QT interval and late I_Na_.^135^ Activation of PI3K translocates PDK1 to the plasma membrane which then activates atypical PKC isoforms and serum and glucocorticoid inducible kinase (SGK).^132^ PDK1 is an important member of the AGC family of protein kinases and it acts an upstream protein kinase to several members of AGC family, including SGK. Mice with conditional knockout of PDK1 exhibit lower heart rate with QRS and QTc interval prolongation, due to reduction of peak I_Na_ and reduced surface expression of sodium channels. Na_V_1.5 channel gating was also changed with a moderate hyperpolarizing shift both in activation and inactivation curves. This decreased surface expression of Na_V_1.5 channel was attributed to PDK1 mediated activation of Foxo1 pathway.^136^

SGK is a serine and threonine kinase, which is transcriptionally regulated by gluco‐ and mineralocorticoids. It is a downstream target of PI3K pathway and phosphorylated by PDK1 which activates SGK.^137^ Three isoforms of this kinase have been identified and named as SGK1, SGK2 and SGK3. Among these, SGK1 and SGK3 are expressed in cardiac tissues and interact with the Na_V_1.5 channel. The co‐expression of SGK1 or SGK3 with Na_V_1.5 channel (hH1 variant) in *Xenopus* oocytes increases I_Na_ and additionally SGK3 also creates a depolarizing shift in the inactivation curve and a hyperpolarizing shift in the activation curve. These effects have been attributed to the phosphorylation of two serine residues at positions 483 and 664 in the Na_V_1.5 channel, identified by mutational analysis (Table 1).^138^, ^139^ SGK1 also controls sodium transport in the kidneys, thus acting as an important contributor in HF and arrhythmia. In pressure overload after transverse aortic constriction (TAC), its acute activation proves to be cardio‐protective however its chronic activation in TAC induced HF acts conversely. Both systolic and diastolic dysfunction has been observed in transgenic mice expressing cardio‐specific, catalytically active SGK1, while transgenic mice expressing the catalytically inactive variant of SGK1 showed normal cardiac structure and function. When additional stress was introduced by TAC, the baseline cardiac dysfunction in catalytically active SGK1 mice was markedly exacerbated, while WT and transgenic mice expressing catalytically inactive SGK1 better tolerated TAC, suggesting that SGK1 inhibition prevents fibrosis, cardiac hypertrophy and development of HF after pressure overload. Moreover, transgenic mice expressing the catalytically active variant of SGK1 exhibit ECG abnormalities, action potential prolongation along with spontaneous ventricular tachycardia. These effects were attributed to increased peak I_Na_, late I_Na_ and changes in Na_V_1.5 channel gating including a hyperpolarizing shift both in the activation and inactivation curves, thus increasing the window current. Surface expression of Na_V_1.5 channels was also increased by SGK1 activation since SGK1 inhibits Nedd_4‐2_, thus preventing Na_V_1.5 channel internalization.^139^ Recently, a threonine residue at position 1590 in the C‐terminal domain of the Na_V_1.5 channel has been described as a candidate site for phosphorylation by SGK1; replacing this threonine with alanine almost completely abolished SGK1‐mediated increase in I_Na_.^140^

Interestingly, PI3K‐mediated effects on I_Na_ appear to be opposite to that of its downstream effector SGK. Moreover Akt and SGK also behave oppositely, suggesting that phosphorylation of the Na_V_1.5 channel is the underlying cause of this differential modulation. PI3K pathway inhibition prolongs QT interval as observed with nilotinib which increases QT interval through inhibition of PI3K pathway.^133^ Besides the Na_V_1.5 channel, PI3K signalling affects several cardiac ion channels,^132^ rendering description of its effect on cardiac electrophysiology complicated and warranting further studies to describe its specific role.

## 5 CONCLUDING REMARKS

Cardiac disorders such as structural heart diseases and arrhythmogenic conduction defects are major public health problems both in developed and developing countries. Use of ion channel blockers can treat arrhythmias but still cannot reduce mortality rates, thus stressing the need for new suitable treatment options. An appealing approach which has emerged recently is antagonizing the protein kinases implicated in pathology of the cardiac disease. One such example is CaMKII which is upregulated in HF and hypertrophy and pharmacological inhibition of which in experimental animal models has proven to be cardio‐protective in a setting of arrhythmias and HF. Kinases and their mediated pathways are also gaining attention as biomarkers for early detection of cardiac disorders. Phosphorylation and dephosphorylation reactions are carried out by more than 500 kinases and 100 phosphatases. Mass‐spectrometry based proteomic studies are unveiling information regarding phosphorylated residues but still a lot of information is missing regarding the sites, sequences, order of phosphorylation, cross talk between the modified residues and how these complicated events unfold to modulate physiological or pathological processes. This missing information strongly requires a concerted effort in the form of future studies to address these questions in order to understand these pathways for the discovery of new drug targets.

## CONFLICT OF INTEREST

The authors declare no conflict of interest.
